# Rupture Risk of Intracranial Aneurysm and Prediction of Hemorrhagic Stroke after Liver Transplant

**DOI:** 10.3390/brainsci11040445

**Published:** 2021-03-31

**Authors:** Hye-Mee Kwon, In-Gu Jun, Kyoung-Sun Kim, Young-Jin Moon, In Young Huh, Jungmin Lee, Jun-Gol Song, Gyu-Sam Hwang

**Affiliations:** Department of Anesthesiology and Pain Medicine, Laboratory for Cardiovascular Dynamics, Asan Medical Center, University of Ulsan College of Medicine, Seoul 05505, Korea; hyemee.kwon@amc.seoul.kr (H.-M.K.); igjun@amc.seoul.kr (I.-G.J.); kyoungsun.kim@amc.seoul.kr (K.-S.K.); yjmoon@amc.seoul.kr (Y.-J.M.); inyoung_huh@amc.seoul.kr (I.Y.H.); Ljungmin7478@gmail.com (J.L.); kshwang@amc.seoul.kr (G.-S.H.)

**Keywords:** end-stage liver disease, intracranial aneurysm, mortality, prediction, intracranial hemorrhage

## Abstract

Postoperative hemorrhagic stroke (HS) is a rare yet devastating complication after liver transplantation (LT). Unruptured intracranial aneurysm (UIA) may contribute to HS; however, related data are limited. We investigated UIA prevalence and aneurysmal subarachnoid hemorrhage (SAH) and HS incidence post-LT. We identified risk factors for 1-year HS and constructed a prediction model. This study included 3544 patients who underwent LT from January 2008 to February 2019. Primary outcomes were incidence of SAH, HS, and mortality within 1-year post-LT. Propensity score matching (PSM) analysis and Cox proportional hazard analysis were performed. The prevalence of UIAs was 4.63% (*n* = 164; 95% confidence interval (CI), 3.95–5.39%). The 1-year SAH incidence was 0.68% (95% CI, 0.02–3.79%) in patients with UIA. SAH and HS incidence and mortality were not different between those with and without UIA before and after PSM. Cirrhosis severity, thrombocytopenia, inflammation, and history of SAH were identified as risk factors for 1-year HS. UIA presence was not a risk factor for SAH, HS, or mortality in cirrhotic patients post-LT. Given the fatal impact of HS, a simple scoring system was constructed to predict 1-year HS risk. These results enable clinical risk stratification of LT recipients with UIA and help assess perioperative HS risk before LT.

## 1. Introduction

Hemorrhagic stroke, including subarachnoid hemorrhage after liver transplantation, is a rare yet devastating complication associated with substantially high mortality [1,2,3]. Considering the increased risk of hemorrhagic stroke due to cirrhosis [4,5,6], the impact of potential risk factors such as concomitant unruptured intracranial aneurysms should be thoroughly evaluated in patients undergoing liver transplantation.

Currently, unruptured intracranial aneurysm findings are increasingly being reported due to improvements in radiological techniques and increased screening [7,8,9]. Unruptured intracranial aneurysms may cause significant mortality if they rupture in the perioperative period. Therefore, the perioperative rupture risk of patients with unruptured intracranial aneurysms undergoing high-risk general surgery unrelated to the unruptured intracranial aneurysm treatment is of great interest [10,11,12]. Coating and wrapping of aneurysms are suggested as alternative aneurysm treatments to invasive surgery treatment; however, re-bleeding risk after those procedures was reported to range from 16.8 to 27.7%, which may pose serious problems in patients with liver cirrhosis [13,14]. Furthermore, the dilemma between the relatively high risk of preventive surgical treatment-related fatality (up to 5%) and uncertainty (as low as 0.25%) of unruptured intracranial aneurysm rupture risk has resulted in an increased number of surgical patients with unruptured intracranial aneurysms [9,15,16]. 

The optimal management of patients with unruptured intracranial aneurysms is a great challenge in the perioperative period for liver transplantation. The characteristics of patients with end-stage liver disease, such as decreased coagulation factors, impaired brain blood flow autoregulation, and higher vascular inflammatory status [5,17], combined with unstable hemodynamics and massive bleeding after liver transplantation, may escalate the risk of aneurysm rupture and, subsequently, cause subarachnoid hemorrhage [1,18]. Moreover, restoration to normal pathophysiology in the postoperative period, including blood pressure elevation [19], may also contribute to the risk of aneurysm rupture. Neurologic sequelae after liver transplantation are reported to be high; they may present up to 30% after liver transplantation, mostly interpreted as sequelae of hepatic encephalopathy [20]. Hemorrhagic stroke after liver transplantation is also of great interest since liver transplantation recipients are faced with numerous risk factors, as mentioned above. The frequency of hemorrhagic stroke after liver transplantation is reported to be 1–3%, with a higher Model for End-stage Liver Disease score (MELDs) and a history of stroke as reported risk factors [20]. Despite the anticipated risks, the prevalence of unruptured intracranial aneurysms is ill-defined, and there have been limited reports on the perioperative risk of subarachnoid hemorrhage from aneurysmal rupture of unruptured intracranial aneurysms and hemorrhagic stroke in patients undergoing liver transplantation. 

Therefore, in the current study, we report the overall prevalence of unruptured intracranial aneurysms in patients with end-stage liver disease and investigate the risk of 1-year and overall subarachnoid hemorrhage and hemorrhagic stroke following postoperative liver transplantation. Post-liver transplantation outcomes between patients with and without unruptured intracranial aneurysms were compared. Furthermore, we investigated risk factors, including the presence of an unruptured intracranial aneurysm and post-liver transplantation 1-year hemorrhagic stroke, and constructed a prediction model that can be used to prognosticate post-liver transplantation 1-year hemorrhagic stroke. 

## 2. Materials and Methods

### 2.1. Patients

Data of recipients who underwent liver transplantation from January 2008 to February 2019 in a tertiary academic hospital in South Korea were collected via a computerized data recording system (Asan Biomedical Research Program, Seoul, South Korea). For the prevalence analysis, the exclusion criteria were as follows: age < 18 years (*n* = 227), patients without preoperative neurovascular imaging (*n* = 626), and preoperative intracranial hemorrhage found in preoperative evaluation (*n* = 28). For the subarachnoid hemorrhage risk analysis, those with unruptured intracranial aneurysms at the time of liver transplantation were included, and those who underwent pre-liver transplantation treatment of unruptured intracranial aneurysms (*n* = 17) were excluded; however, patients with residual aneurysms after unruptured intracranial aneurysm treatment (*n* = 5) were enrolled. This study was approved by the Institutional Review Board of Asan Medical Center, Seoul, Korea, which waived the requirement for written informed consent because of the retrospective study design. The clinical trial number is 2020-0396. This study was conducted in accordance with the ethical standards of the 1975 Helsinki Declaration. No donor organs were obtained from executed prisoners or other institutionalized persons.

### 2.2. Unruptured Intracranial Aneurysm Evaluation

As part of the routine preoperative workup for liver transplantations, neuroimaging studies were performed. An unruptured intracranial aneurysm was diagnosed if intracranial saccular or broad-based aneurysms were found using magnetic resonance angiography, high-resolution three-dimensional computed tomography angiography, or digital subtraction or conventional angiography. If multiple imaging modalities were used, digital subtraction or conventional angiography results were considered first, followed by computed tomography angiography and then magnetic resonance angiography results. Patients suspected of having unruptured intracranial aneurysms from the preoperative radiological evaluation were referred to neurosurgeons and/or stroke neurologists to determine the treatment strategy for the unruptured intracranial aneurysm. Those who underwent pre-liver transplantation surgical treatment of unruptured intracranial aneurysms were excluded unless residual unruptured intracranial aneurysms were found. In patients with multiple aneurysms, the largest unruptured intracranial aneurysm size was used for the per-patient analysis. Unruptured intracranial aneurysm characteristics were evaluated based on formal radiologic reports by our institutional board-certified neuroradiologists and then reviewed by our researcher (S.G.J.).

All unruptured intracranial aneurysm characteristics were stratified according to previous unruptured intracranial aneurysm rupture risk models. Unruptured intracranial aneurysm size was categorized with cut-offs of <3.0, 3.0–6.9, 7.0–9.9, 10–19.9, and ≥20 mm. Unruptured intracranial aneurysm location was classified as anterior cerebral artery, anterior communicating artery, basilar artery, basilar-superior cerebellar artery, internal carotid artery, internal carotid-posterior communicating artery, middle cerebral artery, vertebral artery, or posterior inferior cerebellar artery/vertebrobasilar junction. A daughter sac was defined as an irregular protrusion of the aneurysm wall. Furthermore, the previously reported rupture risk scores of unruptured intracranial aneurysms in the general population (PHASES [21] and Unruptured Cerebral Aneurysm Study of Japan (UCAS Japan) [22]) were computed to estimate the unruptured intracranial aneurysm rupture risk of the current cohort. Given the similarities between Korean and Japanese patients with respect to aneurysm rupture risk [23], Japan was used as the geographic factor when calculating the PHASES score. 

### 2.3. Outcome Measures

The primary outcome was 1-year symptomatic subarachnoid hemorrhage after liver transplantation. Symptomatic subarachnoid hemorrhage was defined as radiologically confirmed subarachnoid bleeding accompanying headache and/or rapidly developing neurological signs or symptoms not due to trauma. The secondary outcome was 1-year and overall hemorrhagic stroke, defined as a composite of intracerebral hemorrhage, intraventricular hemorrhage, subarachnoid hemorrhage, or non-traumatic subdural hematoma. The follow-up period started from the day of liver transplantation and ended either when the patient suffered a corresponding outcome, died, or on 29 February 2020, whichever was first. The entire cohort was followed up for at least 1 year.

### 2.4. Statistical Analysis

Continuous variables are described as mean ± standard deviation (SD) or median and interquartile range (IQR) according to normality, and categorical variables are described as frequencies (%). To compared between two groups, Student’s t-test was performed with continuous variables and chi-square tests were used with categorical variables. To minimize potential confounding effects caused by unseen differences in baseline characteristics, propensity score (PS) matching was performed between patients with and without unruptured intracranial aneurysms. PS were generated through a logistic regression analysis including all of the demographic variables in Table 1. A Poisson rate estimate was used to calculate unruptured intracranial aneurysm prevalence and subarachnoid hemorrhage incidence (details in Supplementary Material 1). Cumulative 1-year and overall subarachnoid hemorrhage, hemorrhagic stroke, and mortality risks were assessed with Kaplan–Meier curves.

To evaluate whether the presence of an aneurysm is a risk factor for post-liver transplantation 1-year hemorrhagic stroke and to identify other risk factors, a Cox proportional regression analysis was performed. Missing values (6, 99, and 18 patients had missing fibrinogen, antithrombin III, and C-reactive protein data, respectively) were filled with multiple imputations of predictive mean matching for the regression analysis. To obtain robustness for variable selection, a relative selection frequency based on a bootstrap resampling method was used for variables with *p* < 0.1 in the univariate analysis. Specifically, we fitted an automated backward variable selection with respect to the Cox proportional hazards model and computed the relative selection frequency for 1000 bootstrap samples. Variables with a relative frequency of >50% were chosen as candidate risk factors. The final prediction model was simplified with a backward selection procedure (excluded if *p* > 0.10), and risk scores according to final risk factors were designated (details in Supplementary Material 2). The risk of 1-year hemorrhagic stroke was calculated using the baseline survival function as follows: Risk estimate = 1 − S_365_^exp(β×risk score)^, where S_365_ (0.9945) is the baseline survival function at 1 year that corresponds to the probability of not experiencing hemorrhagic stroke when all covariates are zero, and β (0.5128) reflects the increase in associated risk. 

We performed Schoenfeld residual testing and visually inspected the log-minus-log plot for each predictor to detect any deviations from the assumption of proportional hazards. Discrimination of the model was examined using Harrell’s concordance (c) statistic, and calibration was examined with a calibration plot and the Greenwood–Nam–D’Agostino calibration test [24] (details in Supplementary Material 3). As prognostic models derived from multivariable regression analyses may overestimate when applied in new patients, a shrinkage estimate was calculated to quantify the optimism. For internal validation, bootstrapping techniques (rather than cross-validations) were performed to estimate the bias-corrected c statistic (Supplementary Material 4), as the outcome incidence was too small for subgroups. *p* values < 0.05 were considered significant. Data manipulation and analyses were performed using R software version 3.6.2 (R Foundation for Statistical Computing) or SAS version 9.4 (SAS Institute; Cary, NC, USA). 

## 3. Results

### 3.1. Patient Characteristics

Of 4425 liver transplantation beneficiaries, 3544 were enrolled in the prevalence analysis after excluding patients who fit the exclusion criteria. The patients’ mean age was 52.8 [9.1] years and 26.9% (*n* = 953) were women. The prevalence of unruptured intracranial aneurysms was 4.63% (*n* = 164; 95% confidence interval (CI), 3.95–5.39%). The mean age of patients with unruptured intracranial aneurysms was 54.0 ± 7.9 years and 39.5% were women. In patients with unruptured intracranial aneurysms, a significantly higher proportion were women (*p* < 0.001) and had a history of hypertension (*p* = 0.017), but the degree of liver cirrhosis severity, history of diabetes, smoking, and dyslipidemia were not different compared with those without unruptured intracranial aneurysms (Table 1). Additional laboratory and intraoperative parameters are presented in Supplementary Table S1. The prevalence of unruptured intracranial aneurysms did not differ across the etiologies of cirrhosis. Among patients with preoperative unruptured intracranial aneurysms, 17 underwent preoperative surgical repair (open or endovascular method) of an aneurysm and were excluded from the subarachnoid hemorrhage risk analysis, but five patients who had residual aneurysms were included, resulting in 3527 patients in the subarachnoid hemorrhage risk analysis (Supplementary Figure S1).

### 3.2. Characteristics of Unruptured Intracranial Aneurysms 

Table 2 shows the characteristics of the unruptured intracranial aneurysms of 147 patients with unruptured intracranial aneurysms at the time of liver transplantation. 

Nearly 90% of the unruptured intracranial aneurysms were <7 mm. The predicted risk of rupture for most unruptured intracranial aneurysms was low (PHASES ≤ 4 or UCAS Japan score ≤ 3). During the follow-up, 21 patients underwent surgical repair of an aneurysm at a median of 6.8 months (range, 4.0–11.0) after liver transplantation (33.3% within 6 months and 81.0% by 1 year). Patients who underwent surgical repair had an aneurysm that was larger or found in a high-risk location or had higher risk scores compared with those without treatment.

### 3.3. Postoperative Outcomes of Patients with and without Unruptured Intracranial Aneurysms

**Figure 1:**
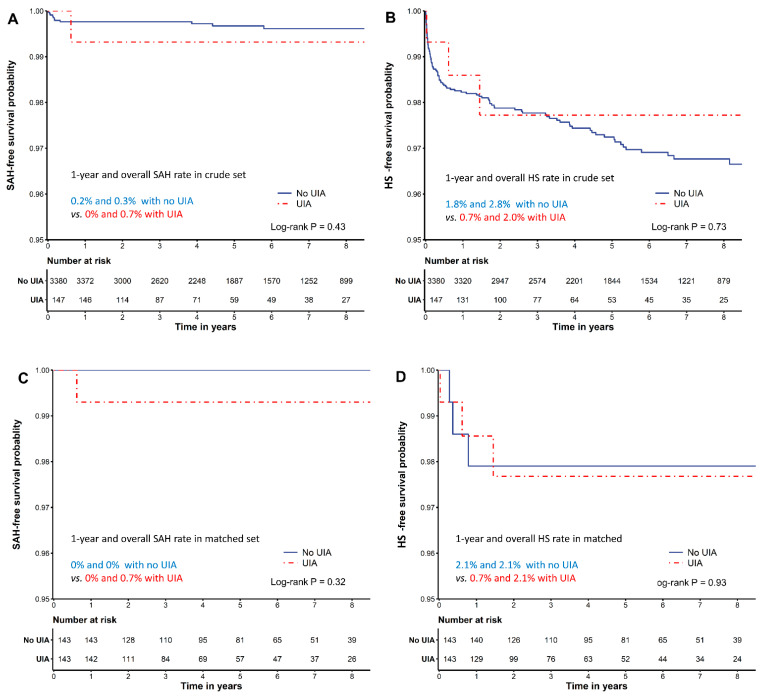
Kaplan–Meier curve showing cumulative (**A**,**C**) subarachnoid hemorrhage-free and (**B**,**D**) hemorrhagic stroke-free survival in crude (**A**,**B**) and propensity score-matched cohorts (**C**,**D**).

During the median follow-up period of 4.5 years (range, 2.2–7.4), only one (0.68%; 95% CI, 0.02–3.79%) of the 147 patients with an unruptured intracranial aneurysm developed symptomatic subarachnoid hemorrhage. Subarachnoid hemorrhage-free survival was not different between patients with and without unruptured intracranial aneurysms (Figure 1).

Overall, postoperative 1-year and overall incidences of hemorrhagic stroke after liver transplantation were 1.7% (*n* = 61; 95% CI, 1.3–2.2%) and 2.8% (*n* = 98; 95% CI, 2.3–3.4%), respectively. The median hemorrhagic stroke-free survival time was 5.4 (range, 3.0–8.1) years. Between those with and without unruptured intracranial aneurysms, the incidences of 1-year hemorrhagic stroke (0.7% versus 1.8%; *p* = 0.501) and overall hemorrhagic stroke (2.0% versus 2.8%; *p* = 0.764) were not significantly different (Supplementary Table S2, Figure 1). In subgroups according to MELDs of 0–19, 20–39, and ≥40, the 1-year incidence rates of hemorrhagic stroke were 2.1%, 3.6%, and 6.3%, respectively, showing a higher incidence of hemorrhagic stroke with higher MELDs.

There were no significant differences in 90-day, 1-year, and overall mortality after liver transplantation before and after PS matching (Supplementary Table S2).

### 3.4. Risk Factors and Prediction Model of 1-Year Hemorrhagic Stroke

We further developed a risk score to predict the 1-year hemorrhagic stroke probability. On univariate analysis with 1-year hemorrhagic stroke as the outcome, the presence of unruptured intracranial aneurysms was not associated with hemorrhagic stroke development within 1-year post-liver transplantation; however, entered the bootstrap model (Supplementary Table S3). After a 1000 bootstrap resampling of the 1-year hemorrhagic stroke-free survival models, variables with a relative selection frequency >50% were MELDs (<20, 20–39, ≥40), thrombocytopenia (platelet count ≤ 50,000 uL^−1^), inflammation (C-reactive protein ≥ 1.8 mg dL^−1^), and history of subarachnoid hemorrhage (Supplementary Table S4). After multivariable Cox regression analysis with backward stepwise selection, these variables were selected as the final risk factors. Table 3 shows the coefficient, adjusted hazard ratios (HRs) of the final model, and the designated scores.

**Figure 2:**
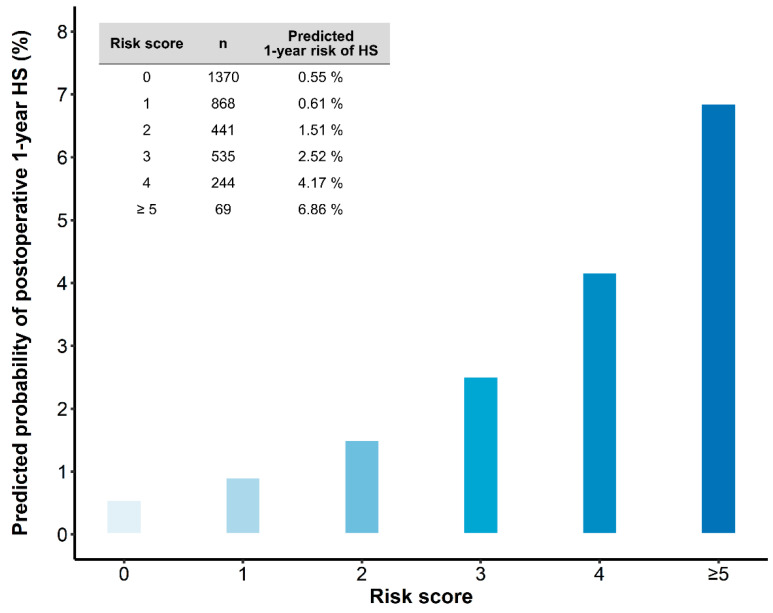
The predicted 1-year risk of hemorrhagic stroke according to the developed risk score. Risk score can be calculated according to the variables in Table 3.

To calculate the risk score for an individual, the number of points associated with each indicator can be added up to obtain the total risk score. The predicted rupture risks of summed risk score are shown in Figure 2.

Schoenfeld residual testing did not reject the proportional hazard assumption of each risk factor. The discrimination of the model for predicting 1-year hemorrhagic stroke probability evaluated by the c statistic was 0.73 (95% CI, 0.67–0.80), indicating good discrimination. The calibration plot showed a good correlation between the predicted and observed probability of 1-year hemorrhagic stroke (Greenwood-Nam-D’Agostino calibration test; chi-square, 6.767, df = 5, *p* = 0.2385; Supplementary Figure S2). The shrinkage estimate was 0.907, indicating that overfitting was not a concern in this model. The 1-year hemorrhagic stroke probability according to risk scores is shown in Figure 2. Our score can be used in combination with Figure 2 to derive predictions for individual patients.

Finally, internal validation with 10,000 bootstraps showed that the bias-corrected c statistic was 0.72 (95% CI, 0.66–0.79), which supports the validity of the proposed scoring system.

## 4. Discussion

This large-scale cohort study demonstrated an unruptured intracranial aneurysm prevalence rate of 4.63% and a 1-year subarachnoid hemorrhage incidence post-liver transplantation in those with unruptured intracranial aneurysms of 0.68%; these values are lower than those of the general population (0.95–1.4%) [21,25]. The presence of asymptomatic unruptured intracranial aneurysms was not associated with postoperative 1-year and overall subarachnoid hemorrhage, hemorrhagic stroke, and mortality. We identified MELDs, thrombocytopenia, inflammation, and history of subarachnoid hemorrhage as risk factors for postoperative 1-year hemorrhagic stroke and developed a simple risk score to predict 1-year hemorrhagic stroke after liver transplantation.

Asymptomatic unruptured intracranial aneurysms detected during preoperative work-up pose a great dilemma for patients and physicians. Although post-liver transplantation aneurysm rupture is a devastating complication and is associated with high mortality, accumulating evidence of the low risk of unruptured intracranial aneurysm rupture and the relatively high morbidity of preventive surgical repair has complicated decision-making for optimum patient management. Additional cirrhosis-related complication risks, such as coagulopathy and thrombocytopenia, further complicate decision-making in the perioperative management of cirrhotic patients prior to liver transplantation [26,27]. Therefore, unruptured intracranial aneurysms’ prevalence and rupture risks in patients undergoing liver transplantation need to be better understood.

Among our patients with liver cirrhosis, 4.63% had unruptured intracranial aneurysms, which was higher than that of the general population without comorbidity (3.2%) [28] and lower than that of patients undergoing cardiovascular surgery (7.26%) [29]. This discrepancy may be due to the unique characteristics of end-stage liver disease. Inflammatory pathological changes in the vascular wall weaken the cerebral arterial wall and contribute to aneurysm formation; these are frequently found in end-stage liver disease patients, resulting in a higher prevalence of unruptured intracranial aneurysms than that in the general population. However, hypertension and hemodynamic sheer stress are also major contributors to aneurysm formation, and low systemic vascular resistance in end-stage liver disease patients is protective, resulting in a lower unruptured intracranial aneurysm prevalence than that in patients who underwent cardiovascular surgery [30].

Previous reports suggested that liver cirrhosis is an independent risk factor for aneurysmal subarachnoid hemorrhage due to liver fibrosis-related cerebral small vessel disease, abnormal systemic vascular tone, vascular malformation, and coagulopathy [17,31]. Furthermore, liver transplantation is a major surgery [32] with dynamic hemodynamic events such as inferior vena cavaclamping and postreperfusion syndrome and is often accompanied by massive bleeding. This may further contribute to aneurysm rupture risk [1,18]. Restoration to normal physiology and manifesting masked predisposing hypertension after liver transplantation may further contribute to aneurysm rupture risk [19]. We anticipated that the risk of aneurysm rupture may be higher in patients with liver cirrhosis undergoing liver transplantation than in the general population. Surprisingly, however, we observed that the risk of aneurysmal subarachnoid hemorrhage was not higher than that in the general population. In fact, this finding is concordant with previous studies demonstrating that unruptured intracranial aneurysm rupture risk did not increase in patients undergoing cardiovascular surgery or in pregnant patients during delivery (these populations are assumed to have a higher unruptured intracranial aneurysm rupture risk) [11,29]. Our findings and previous results consistently indicate that the impact of short-term medical events may not influence unruptured intracranial aneurysm rupture.

Thrombocytopenia, inflammation, and history of subarachnoid hemorrhage, which were established as hemorrhagic stroke risk factors in our prediction model, have also been reported in previous studies [1,2,33,34,35]. Our prediction model showed a significant association of cirrhosis severity, reflected by MELDs, with hemorrhagic stroke risk. Cirrhosis-associated abnormal hemostasis and coagulation due to decreased platelet count and function, decreased levels of clotting factors, fibrinogen abnormality, and vitamin K deficiency may contribute to higher bleeding tendency and may elevate hemorrhagic stroke risk [36]. Furthermore, cerebral autoregulation impairment, which has been associated with cirrhosis severity, may augment hemorrhagic stroke risk [37]. A hemodynamically unstable state at the reperfusion phase of the graft during liver transplantation may expose patients at greater risk due to abrupt changes in perfusion of brain [38]. Increased cerebral blood flow after liver transplantation in patients with chronically impaired cerebral autoregulation could cause large increases in cerebral blood pressure, exposing patients at risk of cerebral hyper-perfusion and triggering hemorrhagic stroke.

The previously reported incidences of hemorrhagic stroke after liver transplantation ranged between 3.9 and 6.7% [1,2,39], which are significantly higher than that reported in the present study (1.7%). This discrepancy may be due to a higher proportion of patients with low MELDs (median, 14) than of those with high MELDs (median, 23–35) in our cohort compared to previous cohorts [1]. A subgroup of patients with MELDs ≥ 40 in the current cohort had an incidence rate of 6.3% for 1-year hemorrhagic stroke, which was similar to that of previous reports. The dose-dependent relationship between cirrhosis severity (assessed by MELDs) and hemorrhagic stroke incidence demonstrates the significant impact of cirrhosis severity on hemorrhagic stroke risk, although a specific mechanism still needs to be elucidated [5,6,40]. Nonetheless, our large cohort with diverse cirrhosis severities may provide a better overall estimate of hemorrhagic stroke incidence in liver transplantation recipients.

Our study has several limitations. First, since postoperative neurovascular imaging was not performed routinely, instances of asymptomatic subarachnoid hemorrhage or intracranial hemorrhage might have been missed, resulting in an underestimation of rupture risk. Secondly, patients who underwent surgical treatment of unruptured intracranial aneurysms after liver transplantation had a higher rupture risk score. Therefore, aneurysms at high risk of rupture were excluded from the subarachnoid hemorrhage incidence analysis, which may have resulted in a selection bias that affected the calculation of subarachnoid hemorrhage risk. Of note, a similar selection bias was also shown in previous prospective cohort studies and rupture risk prediction models [21]. However, care should be taken in the interpretation of results. Thirdly, this was a single-center study with Korean patients. Although the developed risk score was validated internally, external validation with different ethnicities is needed. Considering risk differences caused by genetic predisposition or healthcare systems, models built for specific countries or regions may enhance the validity of the prediction model.

## 5. Conclusions

Previous reports about the prevalence of unruptured intracranial aneurysms in liver transplant recipients or the rupture risk during liver transplant are scarce. In this large cohort study, the unruptured intracranial aneurysm prevalence rate was 4.63%. The presence of unruptured intracranial aneurysms was not a risk factor for subarachnoid hemorrhage, hemorrhagic stroke, or mortality following liver transplantation. Our proposed prediction model of 1-year hemorrhagic stroke risk after liver transplantation is based on easily available patient characteristics such as MELDs, thrombocytopenia, inflammation, and history of subarachnoid hemorrhage. Our results may be useful for the preoperative risk assessment of aneurysm and determination of hemorrhagic stroke risk in liver transplantation recipients.

## Figures and Tables

**Table 1:** Patient characteristics before and after propensity score (PS) matching analysis.

	Crude Cohort			PS-Matched Cohort			
	No UIA	UIA	*p* Value	No UIA	UIA	*p* Value	SMD
	* n * = 3382 (95.4%)	* n * = 162 (4.6%)		* n * = 143	* n * = 143		
Age, years	52.7 ± 9.1	54.1 ± 7.9	0.028	54.0 ± 8.1	54.4 ± 8.1	0.683	0.048
Sex, (men)	2492 (73.7)	99 (60.4)	<0.001	82 (57.3)	86 (60.1)	0.719	0.057
Body mass index, kg m^−^²	24.1 (21.9–26.5)	24.0 (21.7–26.4)	0.798	23.5 (21.7–26.0)	24.0 (21.6–26.4)	0.671	0.005
MELDs	15 (10–25)	13 (10–24)	0.300	13 (9–20)	13 (10–23)	0.343	0.071
<20	2193 (64.9)	109 (66.5)		105 (73.4)	97 (67.8)		
20–39	938 (27.8)	49 (29.9)		32 (22.4)	41 (28.7)		
≥40	249 (7.4)	6 (3.7)		6 (4.2)	5 (3.5)		
Diabetes	794 (23.5)	43 (26.2)	0.478	39 (27.3)	36 (25.2)	0.788	0.048
Hypertension	588 (17.4)	41 (25.0)	0.017	28 (19.6)	33 (23.1)	0.564	0.085
Systolic blood pressure (SBP)	109 (101–119)	111 (101–120)	0.509	108 (100–117)	110 (101–118)	0.560	0.032
SBP > 130 mmHg	316 (9.3)	13 (7.9)	0.635	11 (7.7)	8 (5.6)	0.635	0.084
Current smoker	343 (10.1)	20 (12.2)	0.476	16 (11.2)	16 (11.2)	1.000	<0.001
Dyslipidemia	608 (18.0)	32 (19.5)	0.695	29 (20.3)	28 (19.6)	1.000	0.018
History of SAH	21 (0.6)	15 (9.1)	<0.001	3 (2.1)	5 (3.5)	0.720	0.085
Etiology of cirrhosis			0.903			0.745	0.091
Viral cirrhosis	2165 (64.1)	104 (63.4)		97 (67.8)	96 (67.1)		
Alcoholic cirrhosis	661 (19.6)	31 (18.9)		17 (11.9)	21 (14.7)		
Others	554 (16.4)	29 (17.7)		29 (20.3)	26 (18.2)		
Combined HCC	1516 (44.9)	76 (46.3)	0.769	72 (50.3)	67 (46.9)	0.636	0.070

Values are expressed as the mean (±SD) or median (interquartile range) for continuous variables, and *n* (%) for categorical variables. HCC, hepatocellular carcinoma; MELDs, model for end–stage liver disease score; SAH, subarachnoid hemorrhage; UIA, unruptured intracranial aneurysm; SMD, standardized mean difference.

**Table 2:** Unruptured intracranial aneurysm (UIA) characteristics.

		No SAH	SAH	Treated	Total
		* n * = 128	* n * = 1	* n * = 18	* n * = 147
Modality	DSA	5 (3.9)	0 (0.0)	1 (5.6)	6 (4.1)
	CT	18 (14.1)	0 (0.0)	9 (50.0)	27 (18.4)
	MRI	105 (82.0)	1 (100.0)	8 (44.4)	114 (77.6)
Multiple UIAs		18 (14.1)	0 (0.0)	4 (22.2)	22 (15.0)
Daughter sac		6 (4.7)	0 (0.0)	2 (11.1)	8 (5.4)
Size, mm *	<3 mm	78 (60.9)	1 (100.0)	2 (11.1)	81 (55.1)
	3–6.9 mm	42 (32.8)	0 (0.0)	9 (50.0)	51 (34.7)
	7–9.9 mm	4 (3.1)	0 (0.0)	4 (22.2)	8 (5.4)
	10–19.9 mm	3 (2.3)	0 (0.0)	3 (16.7)	6 (4.1)
	≥20 mm	1 (0.8)	0 (0.0)	0 (0.0)	1 (0.7)
Location	Anterior cerebral artery	6 (4.7)	0 (0.0)	1 (5.6)	7 (4.8)
	Anterior communicating artery	13 (10.2)	0 (0.0)	6 (33.3)	19 (12.9)
	Basilar artery	4 (3.1)	0 (0.0)	1 (5.6)	5 (3.4)
	Internal carotid artery	72 (56.2)	1 (100.0)	1 (5.6)	74 (50.3)
	Posterior communicating artery	6 (4.7)	0 (0.0)	2 (11.1)	8 (5.4)
	Middle cerebral artery	25 (19.5)	0 (0.0)	6 (33.3)	31 (21.1)
	Vertebral artery	0 (0.0)	0 (0.0)	1 (5.6)	1 (0.7)
	Other	2 (1.6)	0 (0.0)	0 (0.0)	2 (1.4)
PHASES score ^†^	≤4	70 (54.7)	1 (100.0)	1 (5.6)	72 (49.0)
	5–7	47 (36.7)	0 (0.0)	12 (66.7)	59 (40.1)
	8–11	11 (8.6)	0 (0.0)	5 (27.8)	16 (10.9)
UCAS Japan score ^ ‡ ^	≤3	109 (85.2)	1 (100.0)	11 (61.1)	121 (82.3)
	4–5	18 (14.1)	0 (0.0)	6 (33.3)	24 (16.3)
	6–8	1 (0.8)	0 (0.0)	1 (5.6)	2 (1.4)

Values are expressed as the mean (±SD) or median (interquartile range) for continuous variables, and *n* (%) for categorical variables. Compared to patients without SAH, patients who received surgical treatment had aneurysms of a significantly larger size * (*p* < 0.001), higher PHASES scores ^†^ (*p* < 0.001), and higher Unruptured Cerebral Aneurysm Study of Japan (UCAS Japan) scores ^‡^ (*p* = 0.026). ^†^ Risk of rupture within five years according to the PHASES score: ≤4, <1%; 5–7, 1.3–2.45%; 8–11, 3.2–7.2% ^‡^ Risk of rupture within three years according to the UCAS Japan score: ≤3, <1%; 4–5, 1.4–2.3%; 6–8, 3.7–7.6%. CT, computed tomography; DSA, digital subtraction angiography; MRI, magnetic resonance imaging; SAH, subarachnoid hemorrhage; UCAS, Unruptured Cerebral Aneurysm Study; UIA, unruptured intracranial aneurysm.

**Table 3:** Final model with Cox regression analysis of risk factors of 1-year hemorrhagic stroke after liver transplantation.

Risk Factors	Points *	β Coefficient	Adjusted HR [95% CI]	*p*-Value
** MELDs **				
<20	0		1	
20–39	2	1.26	3.51 (1.88–6.53)	0.001
≥40	3	1.74	5.69 (2.62–12.33)	<0.001
** History of SAH **				
No	0		1	
Yes	3	1.63	5.09 (1.24–20.98)	0.024
**Platelets**				
>50,000 dL^−1^	0		1	
≤50,000 dL^−1^	1	0.57	1.78 (1.07–2.94)	0.026
** C-reactive protein **				
<1.8 mg dL^−1^	0		1	
≥1.8 mg dL^−1^	1	0.51	1.67 (0.94–2.96)	0.080

CI, confidence interval; HR, hazard ratio; LT, liver transplantation; MELDs, model for end-stage liver disease score; SAH, subarachnoid hemorrhage. Points *: The risk scores were designated according to the coefficient of the variables in the final Cox proportional hazards model, which was divided by the smallest coefficient value (C-reactive protein, β coefficient = 0.51 in our study) and rounded to the nearest integer of the corresponding coefficient.

## Data Availability

Data are available upon request.

## Supplementary Materials

The following are available online at https://www.mdpi.com/article/10.3390/brainsci11040445/s1.
