# Supplementary material for: Rupture Risk of Intracranial Aneurysm and Prediction of Hemorrhagic Stroke after Liver Transplant
Source: Brain Sci. 2021 Mar 31;11(4):445. doi: 10.3390/brainsci11040445 (PMC8066281; doi:10.3390/brainsci11040445)
Supplement: Supplementary file 1 [file brainsci-11-00445-s001.pdf]

Supplementary Materials

for

Rupture Risk of Intracranial Aneurysm and Prediction of Hemorrhagic Stroke after Liver Transplant

Hye-Mee Kwon Ph.D.[1], In-Gu Jun Ph.D.[1], Kyoung-Sun Kim M.D.[1], Young-Jin Moon M.D.[1], In Young Huh Ph.D.[1], Jungmin Lee B.S.[1], Hwa Jung Kim Ph.D.[2], Jun-Gol Song Ph.D.[1,*], Gyu-Sam Hwang Ph.D.[1]

[1]Department of Anesthesiology and Pain Medicine, Laboratory for Cardiovascular Dynamics, Asan Medical Center, University of Ulsan College of Medicine, 88 Olympic-ro 43-gil, Songpa-gu, Seoul 05505, Republic of Korea

[2]Department of Clinical Epidemiology and Biostatistics, Asan Medical Center, University of Ulsan College of Medicine, 88 Olympic-ro 43-gil, Songpa-gu, Seoul 05505, Republic of Korea

[*]Corresponding author:

Jun-Gol Song, M.D., Ph.D.

Department of Anesthesiology and Pain Medicine, Laboratory for Cardiovascular Dynamics, Asan Medical Center, University of Ulsan College of Medicine, 88 Olympic-ro 43-gil, Songpa-gu, Seoul 05505, Republic of Korea

Phone: +82-2-3010-3869; Fax: +82-2-470-1363; Email: jungol.song@amc.seoul.kr

4 Supplementary Tables

2 Supplementary Figures

4 Supplementary Materials

Supplementary Table. 1 Demographics, laboratory and intraoperative parameters before and after propensity score matching analysis

	Crude cohort			PS matched cohort			
	No UIA<br>N=3382 (95.4%)	UIA<br>N=162 (4.6%)	*P* value	No UIA<br>N=143	UIA<br>N=143	*P* value	SMD
Age ≥70 years	45 (1.3)	1 (0.6)	0.657	2 (1.4)	1 (0.7)	1.000	0.069
Sex, Women	888 (26.3)	65 (39.6)	<0.001	61 (42.7)	57 (39.9)	0.719	0.057
SBP >130 mmHg	316 (9.3)	13 (7.9)	0.635	11 (7.7)	8 (5.6)	0.635	0.084
**Medication**							
B–blocker	1044 (30.9)	55 (33.5)	0.529	42 (29.4)	46 (32.2)	0.701	0.061
Calcium channel blocker	255 (7.5)	15 (9.1)	0.546	10 (7.0)	11 (7.7)	1.000	0.027
ACEI or ARB	144 (4.3)	13 (7.9)	0.042	15 (10.5)	10 (7.0)	0.402	0.124
Statin	186 (5.5)	12 (7.3)	0.416	6 (4.2)	11 (7.7)	0.317	0.148
**Laboratory value**							
Hemoglobin, g/dL	10.4 (8.9-12.3)	9.9 (8.7-11.6)	0.091	10.2 (8.9-12.0)	9.9 (8.8-11.9)	0.540	0.029
Platelet, x10^3 mm³/L	61 (41-93)	61 (42-89)	0.552	59 (40-98)	61 (39-88)	0.792	0.067
Platelet≤50 x10^3 mm³/L	1270 (37.6)	69 (42.1)	0.281	63 (44.1)	64 (44.8)	1.000	0.014
Prothrombin time, INR	1.44 (1.21-1.90)	1.40 (1.19-1.79)	0.218	1.34 (1.18-1.60)	1.39 (1.20-1.72)	0.391	0.004
Fibrinogen, mg/dL (n=3538)	157 (114-204)	160 (114-201)	0.933	160 (120-214)	157 (114-201)	0.385	0.104
Antithrombin, % (n=3445)	44 (27-65)	48 (29-65)	0.489	51 (36-71)	48 (31-67)	0.287	0.123
C-reactive protein, mg/dl (n=3509)	0.3 (0.1-1.1)	0.3 (0.1-0.9)	0.403	0.3 (0.1-0.9)	0.3 (0.1-0.9)	0.697	0.048

C-reactive protein > 1.8 mg/dl (n=3509)	518 (15.4)	18 (11.0)	0.152	16 (11.3)	14 (9.8)	0.815	0.051
**Intraoperative variables**							
Deceased donor transplant	492 (14.6)	16 (9.8)	0.110	14 (9.8)	14 (9.8)	1.000	<0.001
Operation time, mins	756 (674–855)	736 (669–836)	0.130	749 (672-847)	735 (670-836)	0.519	0.154
Postreperfusion syndrome	1962 (58.0)	101 (61.6)	0.414	87 (60.8)	90 (62.9)	0.808	0.043
Biopump	808 (23.9)	34 (20.7)	0.402	31 (21.7)	31 (21.7)	1.000	<0.001
Intraoperative CRRT	424 (12.5)	16 (9.8)	0.349	12 (8.4)	14 (9.8)	0.837	0.049
Use of vasopressor	2644 (78.2)	128 (78.0)	1.000	116 (81.1)	113 (79.0)	0.767	0.053
**Intraoperative transfusion**							
packed RBC, unit	8 (3-16)	8 (4-14)	0.968	8 (4-13)	8 (4-14)	0.566	0.056
Massive transfusion†	1499 (44.3)	71 (43.3)	0.853	58 (40.6)	65 (45.5)	0.474	0.099
packed FFP, unit	9 (4-16)	8 (4-14)	0.470	8 (4-12)	8 (4-14)	0.686	0.012
Cryoprecipitate, unit	10 (0-10)	10 (0-10)	0.510	10 (0-10)	10 (0-10)	0.378	0.068
Apheresis platelet, unit	1 (0-1)	1 (0-1)	0.916	1 (0-1)	1 (0-1)	0.740	0.004

Values are expressed as the mean (±SD) or median (interquartile range) for continuous variables, and n (%) for categorical variables.
Antihypertensive medication*: angiotensin converting enzyme inhibitor or angiotensin receptor blocker.
Massive transfusion†: Intraoperative transfusion of packed red blood cells ≥ 10 units.
aPPT, activated partial thromboplastin time; FFP, fresh frozen plasma; INR, international normalized ratio; MELDs, model for end–stage liver disease score; RBC, red blood cell; UIA, unruptured intracranial aneurysm; SMD, standardized mean difference.

Supplementary Table 2. Outcomes according to prevalence of UIAs

	No UIA	UIA	Crude		PSM–adjusted	
	N=3380	N=147	HR [95% CI]	*P* value	HR [95% CI]	*P* value
**SAH**						
One–year	8 (0.2)	0 (0.0)	NA		1.01 [0.80–1.27]	0.953
Overall	11 (0.3)	1 (0.7)	2.25 [0.29–17.46]	0.438	NA	
**HS**						
One–year	60 (1.8)	1 (0.7)	0.39 [0.05–2.79]	0.346	0.34 [0.04–3.30]	0.355
Overall	95 (2.8)	3 (2.0)	0.82 [0.26–2.57]	0.727	1.06 [0.21–5.27]	0.941
**Mortality**						
90–day	112 (3.3)	4 (2.7)	0.81 [0.30–2.21]	0.687	0.67 [0.19–2.36]	0.529
One–year	259 (7.7)	7 (4.8)	0.61 [0.29–1.30]	0.202	0.43 [0.18–1.04]	0.062
Overall	498 (14.7)	13 (8.8)	0.63 [0.36–1.10]	0.103	0.66 [0.32–1.37]	0.269

Values are expressed as n (%)
UIA, unruptured intracranial aneurysm; HR, hazard ratio; CI, confidence interval; PSM, propensity score matching; SAH, subarachnoid hemorrhage; HS, hemorrhagic stroke.

Supplementary Table 3. Univariate Cox regression analysis of demographics and preoperative variables associated with 1–year hemorrhagic stroke risk

Variables	HR [95% CI]	*P* value
Presence of aneurysm	0.39 [0.05-2.79]	0.346
Age, years	1.02 [0.99-1.05]	0.187
Women	1.06 [0.6-1.85]	0.848
Body mass index, kg/m²	0.94 [0.88-1.02]	0.127
MELDs	1.06 [1.04-1.08]	<0.001
MELDs		
< 20	1 [Reference]	
20–39	4.18 [2.30-7.58]	<0.001
≥ 40	7.63 [3.76-15.47]	<0.001
Diabetes	1.15 [0.65-2.04]	0.628
Hypertension	1.03 [0.54-1.97]	0.934
Systolic blood pressure> 130 mmHg	1.95 [0.99-3.84]	0.054
Current smoker	1.33 [0.63-2.81]	0.447
History of SAH	4.31 [1.05-17.66]	0.042
Dyslipidemia	0.59 [0.27-1.29]	0.184
Etiology of cirrhosis		
Viral cirrhosis	1 [Reference]	
Alcoholic cirrhosis	1.56 [0.86-2.83]	0.141
Others	1.26 [0.64-2.50]	0.499
Combined HCC	0.43 [0.24-0.77]	0.004
B–blocker	0.66 [0.37-1.21]	0.179
ACEI or ARB	0.36 [0.05-2.62]	0.314
Platelet, ≤ 50 x10^3 mm³/L	1.95 [1.18-3.22]	0.009
C-reactive protein ≥ 1.8 mg/dl	3.18 [1.89-5.37]	<0.001

HR, hazard ratio; CI, confidence interval; MELDs, model for end–stage liver disease score.
Massive transfusion*: Intraoperative transfusion of packed red blood cells ≥ 10 units.

Supplementary Table 4. Relative Selection Frequency Based on 1000 Bootstrap Re–Sampling of Cox regression analysis with 1–year intracranial bleeding occurrence as outcome

Variable	Relative frequency
Presence of aneurysm	430
MELDs of < 20, 20-39, ≥40	657
Systolic blood pressure > 130 mmHg	441
History of SAH	603
Combined HCC	316
Platelet ≤ 50 x10^3 mm^3/L	799
C-reactive protein ≥ 1.8 mg/dl	632

MELDs, model for end–stage liver disease score; SAH, subarachnoid hemorrhage; HCC, hepatocellular carcinoma.

Supplementary Figure Legends

Supplementary Figure 1. Patient flow diagram of inclusion and exclusion criteria

Supplementary Figure 2. Calibration plot of observed and predicted 1-year hemorrhage stroke risk

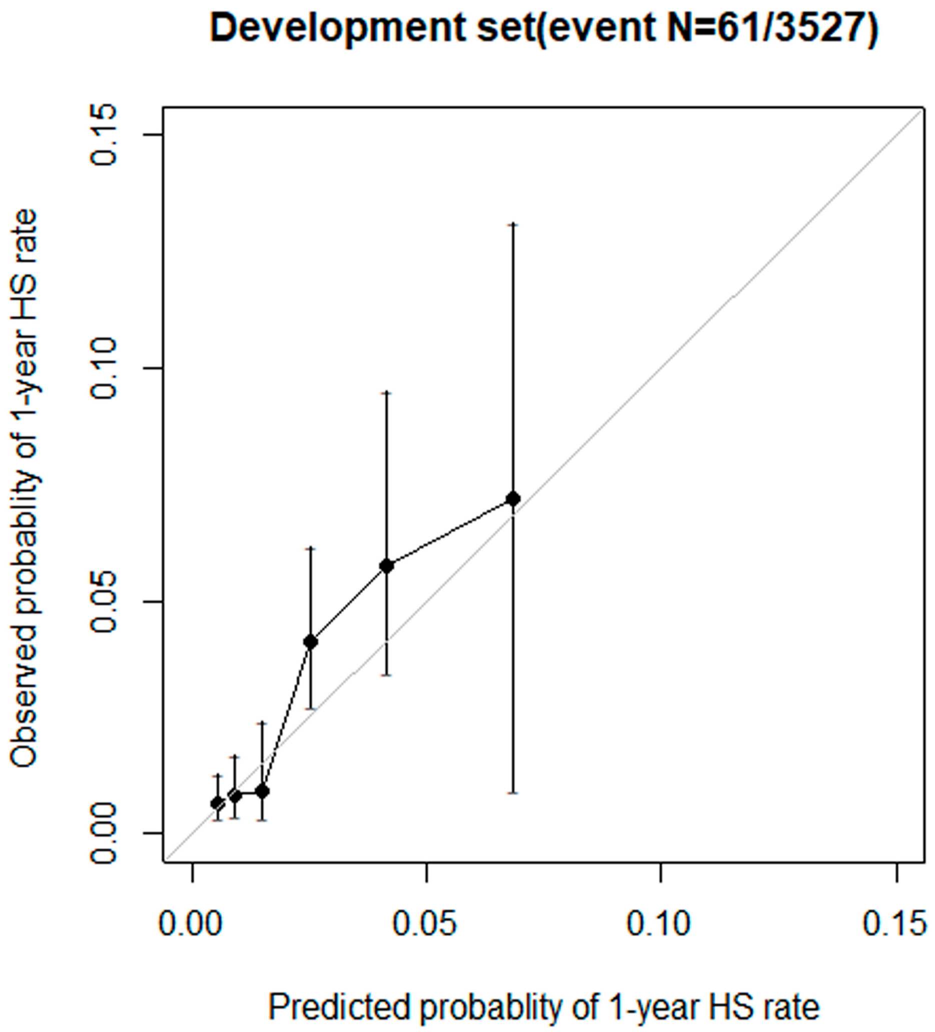

Supplementary material 1.

To reduce the influence of possible unseen bias between those with and without aneurysm, we used the propensity score matching method when comparing the outcome. All demographic variables shown in Table 1 without regard to outcomes were used to obtain the propensity score. Propensity score was estimated with groups as the dependent variable by a multiple logistic regression analysis. Model discrimination was evaluated with Harrell's concordance statistic (0.774), and model calibration was evaluated with Hosmer–Lemeshow statistics (chi-squared, 5.9032; df, 8; P=0.6581). For matching, 1:1 propensity score–matched pairs were created with a caliper of 0.2. We assessed the balance of baseline demographic variables between the two groups with standardized differences for each covariate (Table 1). Continuous variables were compared with the use of the paired t test or Wilcoxon signed-rank test, and categorical variables were compared with the McNemar test, as appropriate. In the propensity score–matched cohort, the risks of each outcome were compared with logistic regression or a Cox regression model, as appropriate.

Supplementary material 2.

The risk scores were designated according to the coefficient of the variables in the final Cox proportional hazards model, which was divided by the smallest coefficient value (c-reactive protein, β coefficient=0.51 in our study) and rounded to the nearest integer of the corresponding coefficient.

Supplementary material 3.

Discrimination of model was examined with Harrell's concordance (c) statistic to measure the performance of the model, which indicates to what extent the model distinguishes the risk of hemorrhagic stroke (HS) during follow-up. To assess calibration of predicted risks and the observed risks, a calibration plot and Greenwood-Nam-D'Agostino calibration test were performed.

Supplementary material 4.

We internally validated the performance of the model by a bootstrapping technique. Simulation studies have shown that this approach provides the least biased and most stable estimates of optimism–corrected performance among the various proposed methods for internal validation, with "optimism" referring to the inherent bias toward an overestimated performance in the derivation dataset. Briefly, optimism in a performance measure (e.g., Harrell's concordance statistic) with this method is estimated by the average of measurements of the bootstrap sample subtracted by measurements of the original data set($measure_{bootstrap\ sample}$ $-measure_{original\ dataset}$) for a large number of models derived from respective 1000 bootstrap samples: the performance of each of the bootstrap sample–derived models is evaluated on the bootstrap sample ("training" dataset) and back to the original dataset ("validation" dataset). The average ($measure_{bootstrap\ sample}$ $-measure_{original\ dataset}$), i.e., the optimism, is then subtracted from the original performance measure (i.e., the Harrell's concordance statistic of the original model) to provide a more realistic estimate. This approach moderates our expectations from the model and sets an upper limit for performance in future external validation.
